# Diagnosing thyroid nodules with atypia of undetermined significance/follicular lesion of undetermined significance cytology with the deep convolutional neural network

**DOI:** 10.1038/s41598-021-99622-0

**Published:** 2021-10-08

**Authors:** Inyoung Youn, Eunjung Lee, Jung Hyun Yoon, Hye Sun Lee, Mi-Ri Kwon, Juhee Moon, Sunyoung Kang, Seul Ki Kwon, Kyong Yeun Jung, Young Joo Park, Do Joon Park, Sun Wook Cho, Jin Young Kwak

**Affiliations:** 1 Department of Radiology, Kangbuk Samsung Hospital, Sungkyunkwan University School of Medicine, Seoul, Republic of Korea; 2 Department of Computational Science and Engineering, Yonsei University, Seoul, Republic of Korea; 3 Department of Radiology, Yonsei University College of Medicine, 50 Yonsei-ro, Seodaemun-gu, Seoul, 03722 Republic of Korea; 4 Biostatistics Collaboration Unit, Yonsei University College of Medicine, Seoul, Republic of Korea; 5 Department of Internal Medicine, Seoul National University College of Medicine, Seoul National University Hospital, 101 Daehak-ro, Jongno-gu, Seoul, 03080 Republic of Korea; 6 Department of Internal Medicine, Nowon Eulji Medical Center, Eulji University, Seoul, Republic of Korea

**Keywords:** Cancer, Cancer imaging, Cancer imaging

## Abstract

To compare the diagnostic performances of physicians and a deep convolutional neural network (CNN) predicting malignancy with ultrasonography images of thyroid nodules with atypia of undetermined significance (AUS)/follicular lesion of undetermined significance (FLUS) results on fine-needle aspiration (FNA). This study included 202 patients with 202 nodules ≥ 1 cm AUS/FLUS on FNA, and underwent surgery in one of 3 different institutions. Diagnostic performances were compared between 8 physicians (4 radiologists, 4 endocrinologists) with varying experience levels and CNN, and AUS/FLUS subgroups were analyzed. Interobserver variability was assessed among the 8 physicians. Of the 202 nodules, 158 were AUS, and 44 were FLUS; 86 were benign, and 116 were malignant. The area under the curves (AUCs) of the 8 physicians and CNN were 0.680–0.722 and 0.666, without significant differences (*P* > 0.05). In the subgroup analysis, the AUCs for the 8 physicians and CNN were 0.657–0.768 and 0.652 for AUS, 0.469–0.674 and 0.622 for FLUS. Interobserver agreements were moderate (k = 0.543), substantial (k = 0.652), and moderate (k = 0.455) among the 8 physicians, 4 radiologists, and 4 endocrinologists. For thyroid nodules with AUS/FLUS cytology, the diagnostic performance of CNN to differentiate malignancy with US images was comparable to that of physicians with variable experience levels.

## Introduction

Thyroid nodules occur commonly with incidence rates going up to 68%^1^, and ultrasonography (US) is the primary screening method used to detect these nodules with high sensitivity and specificity. Fine-needle aspiration (FNA) is an easy, relatively safe, and highly accurate diagnostic tool that can be performed under US-guidance to identify benign and malignant nodules based on US findings.

The Bethesda system is a standardized, category-based reporting system for thyroid cytopathology, and widely used to interpret FNA results^2^. The nodules with Bethesda class III lesions, otherwise known as atypia of undetermined significance (AUS) or follicular lesion of undetermined significance (FLUS), have a malignancy risk of 6–18%, and management plans vary widely from clinical observation, US follow up, repeat FNA or core needle biopsy, molecular test to thyroid surgery^2,3^. Although thyroid US examination has been shown to help stratify the risk of Bethesda class III lesions^3,4^, US assessment is limited in application due to its inherent limitations of poorly reproducible tests^5^.

Recently, machine learning and deep learning methods have been developed, and have rapidly become the methodology of choice for medical image analysis^6,7^. The deep convolutional neural network (CNN) is trained with an automated process using raw image pixels rather than engineered features extracted by experts of the traditional machine learning algorithm^7^. For thyroid cancer diagnosis, many machine learning and deep learning techniques have been implemented^8–12^. When machine learning techniques using support vector machines were compared with an experienced radiologist, they showed lower accuracy^13^, while deep learning techniques showed similar accuracies to experienced radiologists and higher accuracies than inexperienced radiologists^12,14^. Recently, we developed a computer-aided program that uses a deep convolutional neural network (CNN) to diagnose thyroid nodules according to US features^14^. This CNN can be an objective, operator-independent method to identify benign lesions and malignancy, and these advantages are thought to be especially helpful for nodules with AUS/FLUS cytology on FNA in predicting malignant risk and determining the next management step.

The purpose of this study was to compare the diagnostic performances of physicians with varying experience levels and CNN to predict malignancy using US images of thyroid nodules with Bethesda class III results on FNA.

## Results

Table 1 summarized the demographic features of the included 202 nodules. There were 86 (42.6%) benign nodules and 116 (57.4%) malignancies confirmed after surgery. The pathologic results after surgery were shown in Table 2. Of 202 nodules, preoperative FNA found 158 with AUS cytology and 44 with FLUS cytology. There was no statistical difference between the benign and malignant nodules for sex and age. Malignant nodules had significantly smaller size than benign ones (*P* = 0.009), and higher cancer probabilities than benign nodules using CNN (*P* < 0.001).

**Table 1:** Summary of the demographic features.

	Total		Benign		Malignancy		*P* value
Numbers of nodules	202		86 (42.6%)		116 (57.4%)		
**Sex**							0.416
Male	48		18 (20.9%)		30 (25.9%)		
Female	154		68 (79.1%)		86 (74.1%)		
Mean age (years)^a^			47.9 ± 13.3		47.0 ± 14.8		0.669
**Cytologic result**							< 0.001
*No. of AUS*							
Institution A^b^	158	78	50 (58.1%)	23	108 (93.1%)	55	
Institution B^b^		43		14		29	
Institution C^b^		37		13		24	
*No. of FLUS*							
Institution A^b^	44	34	36 (41.9%)	29	8 (6.9%)	5	
Institution B^b^		1		1		0	
Institution C^b^		9		6		3	
Median size (IQR, mm)^c^			19.5 (13–32)		13.5 (11–23)		0.009
Median cancer probability calculated by CNN (IQR, %)^c^			36.5 (18.7–69.5)		67.7 (30.2–89.9)		< 0.001

*AUS* atypia of undetermined significance, *FLUS* follicular lesion of undetermined significance, *IQR* interquartile range, *CNN* deep convolutional neural network.

^a^The independent two sample t-test.

^b^We collected consecutive patients from three institutions, and the numbers of patients recruited from each hospital was expressed as Institution A, B, and C.

^c^The Mann–Whitney U test.

**Table 2:** Pathologic results after surgery.

Pathologic result	AUS	FLUS	Total
**Benign**			
Adenomatous hyperplasia	22 (44.0)	12 (3.3)	34 (39.5)
Follicular adenoma	19 (38.0)	20 (55.6)	39 (45.3)
Hurthle cell adenoma	3 (6.0)	3 (8.3)	6 (7.0)
Noninvasive follicular thyroid neoplasm with papillary-like nuclear feature	3 (6.0)	1 (2.8)	4 (4.7)
Hyaline trabecular tumor	1 (2.0)	–	1 (1.2)
Localized fibrosis	1 (2.0)	–	1 (1.2)
Lymphocytic thyroiditis	1 (2.0)	–	1 (1.2)
*Total*	50	36	86
**Malignancy**			
Papillary thyroid carcinoma	99 (91.7)	4 (50.0)	103 (88.8)
Follicular carcinoma	8 (7.4)	3 (37.5)	11 (9.5)
Poorly differentiated carcinoma	1 (0.9)	1 (12.5)	2 (1.7)
*Total*	108	8	116

Data in parentheses are percentages.

*AUS* atypia of undetermined significance, *FLUS* follicular lesion of undermined significance.

**Figure 1:**
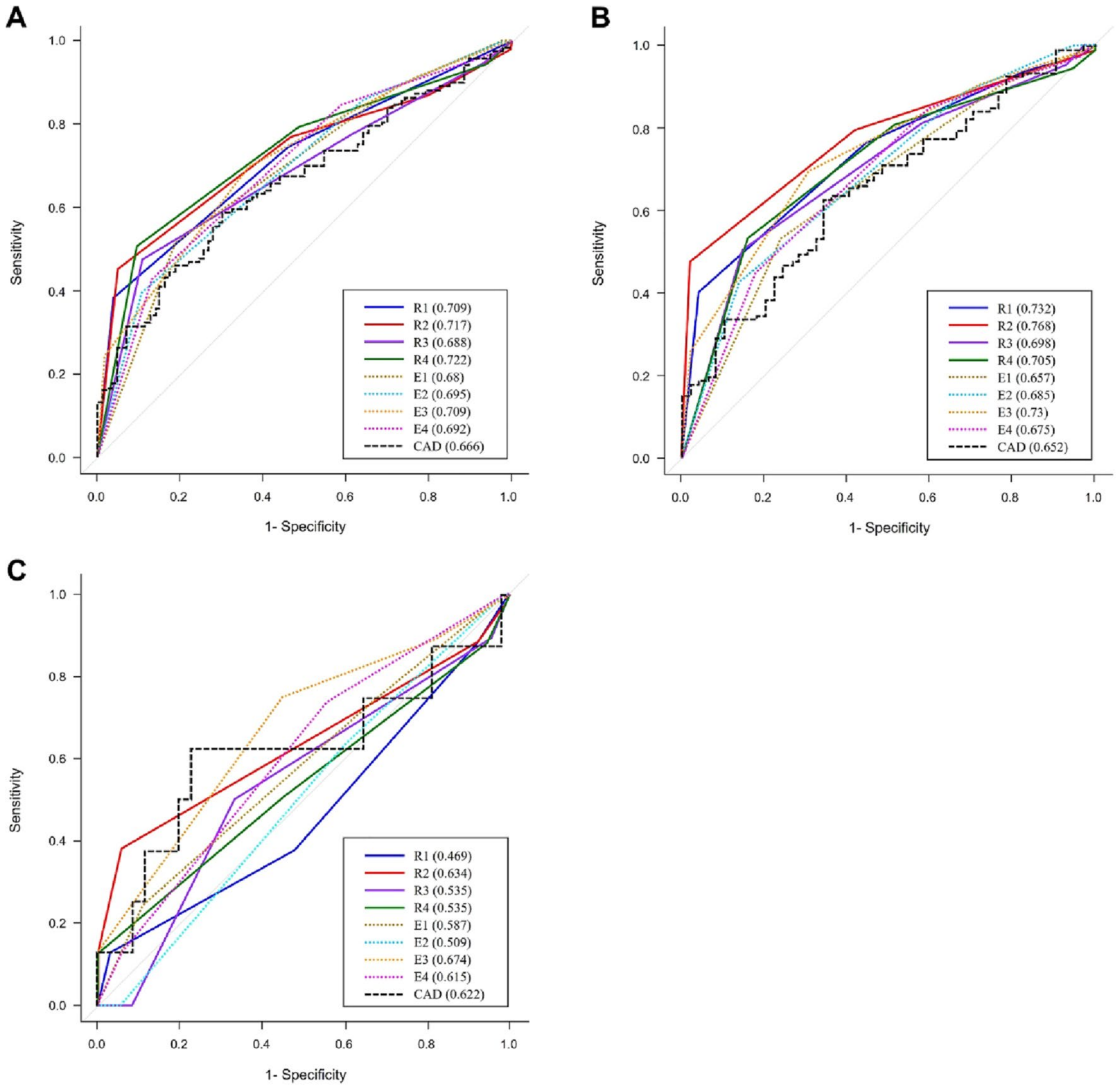
Comparing diagnostic performances between the 8 physicians and CNN using the receiver operating characteristic analysis for the atypia of undetermined significance (AUS)/follicular lesion of undetermined significance (FLUS, **A**), only AUS (**B**), and only FLUS (**C**) groups. Data in parentheses are the AUC results of each physician or CNN. *CNN* deep convolutional neural network, *AUS* atypia of undetermined significance, *FLUS* follicular lesion of undetermined significance, *R* radiologist, *E* endocrinologist.

The diagnostic performances of the 8 physicians and CNN were compared in Table 3. The sensitivity, specificity, and AUC of the 8 physicians were 24.1–50.9%, 81.4–98.8%, and 0.680–0.722, respectively (Table 3, Fig. 1). The calculated sensitivity, specificity, and AUC of CNN were 59.5%, 69.8%, and 0.666, respectively, using an estimated cut-off value of 54.1% (Table 3, Fig. 1). CNN showed significantly higher sensitivity than 6 physicians, but not over Radiologist 4 (50.0%; *P* = 0.082) and Endocrinologist 1 (50.9%; *P* = 0.137). CNN showed significantly lower specificity than all 8 physicians (*P* < 0.05). CNN had similar AUC values compared to the 8 physicians, without statistical difference (*P* > 0.05).

**Table 3:** Diagnostic performances of the 8 physicians and deep convolutional neural network.

	Sensitivity	*P* value^a^	Specificity	*P* value^a^	AUC	*P* value^b^
**Total 202 nodules**						
R1	37.9% (29.1–46.8%)	< 0.001	96.5% (92.6–100%)	< 0.001	0.709 (0.643–0.776)	0.279
R2	44.8% (35.8–53.9%)	0.008	95.3% (90.9–99.8%)	< 0.001	0.717 (0.649–0.784)	0.187
R3	47.4% (38.3–56.5%)	0.020	89.5% (83.1–96.0%)	< 0.001	0.688 (0.62–0.757)	0.568
R4	50.0% (40.9–59.1%)	0.082	90.7% (84.6–96.8%)	< 0.001	0.722 (0.654–0.789)	0.145
E1	50.9% (41.8–60.0%)	0.137	81.4% (73.3–89.6%)	0.015	0.680 (0.612–0.749)	0.742
E2	39.7% (30.8–48.6%)	0.001	89.5% (83.1–96.0%)	0.001	0.695 (0.629–0.760)	0.500
E3	24.1% (16.4–31.9%)	< 0.001	98.8% (96.6–100%)	< 0.001	0.709 (0.642–0.775)	0.305
E4	42.2% (33.3–51.2%)	0.001	87.2% (80.2–94.3%)	0.002	0.692 (0.624–0.761)	0.494
CNN	59.5% (50.5–68.4%)		69.8% (60.1–79.5%)		0.666 (0.592–0.740)	
**AUS (n = 158)**						
R1	39.8% (30.6–49.0%)	< 0.001	96.0% (90.6–100%)	< 0.001	0.732 (0.658–0.806)	0.111
R2	47.2% (37.8–56.6%)	0.011	98.0% (94.2–100%)	< 0.001	0.768 (0.699–0.837)	0.011
R3	50.0% (40.6–59.4%)	0.029	86.0% (76.4–95.6%)	0.008	0.698 (0.618–0.778)	0.336
R4	52.8% (43.4–62.2%)	0.110	84.0% (73.8–94.2%)	0.008	0.705 (0.624–0.786)	0.253
E1	52.8% (43.4–62.2%)	0.128	76.0% (64.2–87.8%)	0.123	0.657 (0.574–0.741)	0.913
E2	42.6% (33.3–51.9%)	0.001	86.0% (76.4–95.6%)	0.008	0.685 (0.605–0.765)	0.525
E3	25.0% (16.8–33.2%)	< 0.001	98.0% (94.2–100%)	< 0.001	0.730 (0.654–0.806)	0.110
E4	44.4% (35.1–53.8%)	0.002	82.0% (71.4–92.6%)	0.037	0.675 (0.59–0.759)	0.628
CNN	62.0% (52.9–71.2%)		66.0% (52.9–79.1%)		0.652 (0.563–0.741)	
**FLUS (n = 44)**						
R1	12.5% (0–35.4%)	0.046	97.2% (91.9–100%)	0.011	0.469 (0.234–0.703)	0.435
R2	12.5% (0–35.4%)	0.046	91.7% (82.6–100%)	0.119	0.634 (0.372–0.895)	0.902
R3	12.5% (0–35.4%)	0.046	94.4% (87.0–100%)	0.046	0.535 (0.313–0.757)	0.493
R4	12.5% (0–35.4%)	0.046	100% (100–100%)	0.001	0.535 (0.290–0.780)	0.699
E1	25.0% (0–55.0%)	0.128	88.9% (78.6–99.2%)	0.239	0.587 (0.371–0.803)	0.857
E2	0% (0–0%)	0.001	94.4% (87.0–100%)	0.046	0.509 (0.320–0.697)	0.528
E3	12.5% (0–35.4%)	0.046	100% (100–100%)	0.001	0.674 (0.465–0.882)	0.803
E4	12.5% (0–35.4%)	0.046	94.4% (87.0–100%)	0.046	0.615 (0.420–0.809)	0.970
CNN	62.5% (29.0–96.0%)		77.8% (64.2–91.4%)	0.808	0.622 (0.355–0.888)	

*R* radiologist, *E* endocrinologist, *CNN* deep convolutional neural network, *AUS* atypia of undetermined significance, *FLUS* follicular lesion of undetermined significance.

^a^Compared with the results of the convolutional neural network (CNN) using by generalized estimating equation.

^b^Compared with the results of the CNN using by DeLong’s test.

In the 158 nodules of the AUS group, the sensitivity, specificity, and AUC of the 8 physicians ranged 25.0–52.8%, 76.0–98.0%, and 0.657–0.768, respectively, while the sensitivity, specificity, and AUC value of CNN was 62.0%, 66.0%, and 0.652 with a cut-off value of 54.1% (Table 3, Fig. 1). CNN showed significantly higher sensitivity than 6 physicians (ranges, 25.0–50.0%; *P* < 0.05) but not over Radiologist 4 (52.8%; *P* = 0.110) and Endocrinologist 1 (52.8%; *P* = 0.128). CNN showed significantly lower specificity than 7 physicians (ranges, 82.0–98.0%; *P* < 0.050), but not lower than Endocrinologist 1 (76.0%; *P* = 0.123), and CNN had relatively lower AUC values than all 8 physicians, but this difference was only significant in Radiologist 2 (*P* = 0.011).

In the 44 nodules of the FLUS group, the sensitivity, specificity, and AUC of the 8 physicians were 0–25.0%, 88.9–100%, and 0.469–0.674, respectively. The sensitivity, specificity, and AUC value of CNN was 62.5%, 77.8%, and 0.622, respectively, with an estimated cut-off value of 15.9% (Table 3, Fig. 1). CNN showed significantly higher sensitivity than 7 physicians (ranges, 0–12.5%; *P* < 0.050) but not over Endocrinologist 1 (25.0%; *P* = 0.128). CNN showed significantly lower specificity than 6 physicians (*P* < 0.050) but not lower than Radiologist 2 (91.7%, *P* = 0.119) and Endocrinologist 1 (88.9%, *P* = 0.239). AUC values did not differ between the 8 physicians and CNN (*P* > 0.050).

For interobserver variability, the 8 physicians showed moderate agreement (k = 0.543; 95% confidence interval [CI], 0.381–0.414), the 4 radiologists substantial agreement (k = 0.652; 95% CI, 0.596–0.709), and the 4 endocrinologists moderate agreement (k = 0.455; 95% CI, 0.399–0.511). In the subgroup analysis for the 158 nodules with AUS cytology, the 8 physicians showed moderate agreement (k = 0.523; 95% CI, 0.493–0.552), the 4 radiologists substantial agreement (k = 0.624; 95% CI, 0.560–0.687), and the 4 endocrinologists moderate agreement (k = 0.447; 95% CI, 0.383–0.511). The 8 physicians showed fair agreement (k = 0.349; 95% CI, 0.293–0.405), substantial agreement (k = 0.647; 95% CI, 0.526–0.767), and slight agreement (k = 0.106; 95% CI, 0.015–0.226) for the 44 nodules with FLUS cytology.

## Discussion

The AUS/FLUS cytology includes a heterogeneous and broad spectrum of diagnoses which contain more pronounced cells with architectural and/or nuclear atypia than benign lesions but not enough of these cells to be considered malignant, and have a malignancy risk of 6–18% after NIFTP is removed which can make it difficult for clinicians to reach a decision on further management^2^. For nodules of this category, we can perform repeat FNA/CNB or molecular tests as supplementary evaluation methods instead of proceeding to surgery; however, even results from repeated FNA show the same cytology in 10–30% of the nodules^15^. In nodules with AUS/FLUS cytology, US features can help stratify the malignancy risk of thyroid nodules^3,4,16–18^. A meta-analysis study showed that the more suspicious US features a nodule has, the more likely it is to be malignant^3^, with similar results being observed in nodules with AUS cytology, but not in those with FLUS cytology^16,17^. However, the US examination itself is highly subjective, operator dependent and less reproducible than other imaging methods^5,19^.

CNN is a typical deep learning algorithm based on feature recognition^9,20,21^. It can extract regular features automatically from 2D images including thyroid US to achieve good diagnostic results; thus, CNN is more objective and highly reproducible compared to US when assisting diagnosis^20,22–25^. Several recent studies have shown comparable diagnostic performance between radiologists and CNN for evaluating thyroid nodules on US^22–25^. This study mainly aimed to suggest a possible supportive role of CNN for predicting malignancy in AUS/FLUS lesions. Past studies have compared the diagnostic performances of CNN and human physicians, but to our knowledge, all of the physicians in these past studies were radiologists^22,24–26^. Our study compared the diagnostic performances of 8 physicians and CNN for diagnosing thyroid malignancy and the physicians in our study were a heterogeneous group of 4 radiologists and 4 endocrinologists with variable levels of experience.

Among the machine learning and deep learning methods newly developed,, CNN showed the highest accuracy and specificity to differentiate Bethesda category III nodules from Bethesda IV/V/VI nodules using US images^27^. This previous study was performed to make decisions on treatment, but diagnostic accuracy was not compared between the clinician and the machine or deep learning approaches. In contrast, both radiologists and endocrinologists with varying levels of experience performed US analyses in our study to predict malignancy in thyroid nodules with AUS/FLUS cytology. We found the AUC of CNN to be similar to those of the 8 physicians for diagnosing malignancy. CNN showed higher sensitivity and lower specificity for diagnosing malignancy in AUS/FLUS lesions than the 8 physicians and these results were comparable to those of other recent studies with higher sensitivity and lower specificity for CNN compared to radiologists^13,22,25,26^. However, our results for both CNN and radiologists showed relatively lower sensitivity, higher specificity, and lower AUC values than other studies^22,25,26^. Our study only included nodules with AUS/FLUS confirmed at FNA. Furthermore, the structures of CNNs are varying in each study and used cut-off values to make the decision based on the probability results from CNNs (there are diverse approaches to determine the cut-off value) are different. In comparison, other studies included thyroid nodules without considering their cytologic results of FNA. Thus, the absolute values of the diagnostic performances are affected by these differences. Rather than weighing the absolute values of the diagnostic performances, it would be more appropriate to check and compare trends. Moreover, most of our study population consisted of AUS nodules (78.2%), and CNN also showed similar diagnostic performances with AUS/FLUS.

Interobserver variability is a very important issue because US is highly subjective and operator dependent as mentioned above, and diagnosis using captured JPEG images is more subjective^5,19^. There was a study evaluating the interobserver variability of three radiologists with various experience levels (a resident, a fellow, and a staff), and moderate agreement was observed for each US characteristic (k = 0.473–0.634) except for shape (k = 0.034)^26^. Ko et al. reported fair interobserver variability between two radiologists using TI-RADS by Kwak et al., and criteria by Kim et al.^25^. We only analyzed risk levels according to the ACR TI-RADS system for interobserver variability, and did not analyze each US feature. Our results showed moderate interobserver variability among the 8 physicians. Substantial agreement was observed between the 4 radiologists, which is slightly superior to the interobserver variability of all 8 physicians and also the interobserver variability of 4 endocrinologists. Our 4 radiologists had different levels of experience with thyroid US, but their daily work exposed them much more to US images, making them also much more familiar with US images and the ACR TI-RADS system than endocrinologists.

Our study has several limitations. First, there was selection bias due to its retrospective study design. Second, the total sample size was not large despite it being a multicenter study, and the number of FLUS cytology nodules was only 44 (21.8%), which is relatively small for generalizing its findings to an entire population. Third, the malignancy rate after surgery was 57.4%, much higher than the rate recommended by the Bethesda system^2^. For AUS/FLUS cytology, excision can be considered when repeated FNA/CNB or molecular tests are not helpful or nodules show suspicious US characteristics. We used the inclusion criteria of surgery-performed lesions only, thus, a higher malignancy rate is expected. Fourth, we only compared the risk levels of the ACR TI-RADS system without considering each US feature, which again was a point of conflict between the 8 physicians (Supplementary Table 1).

The diagnostic performance of CNN was comparable to that of physicians with variable experience levels in differentiating malignancy from thyroid nodules with AUS/FLUS cytology on US.

## Methods

This multicenter study was based on patient data collected from three tertiary referral institutions in South Korea. The institutional review boards (IRB) of all three institutions approved this retrospective observational study and the need of informed consent was waived for the review of patient images and records by three IRBs (Kangbuk Samsung Hospital Institutional Review Board, 2020-03-020; Yonsei University Health System, Severance Hospital, Institutional Review Board, 4-2020-0106; and Seoul National University College of Medicine/ Seoul National University Hospital Institutional Review Board, 1911-039-1076). This study was performed in accordance with relevant guidelines and regulations.

**Figure 2:**
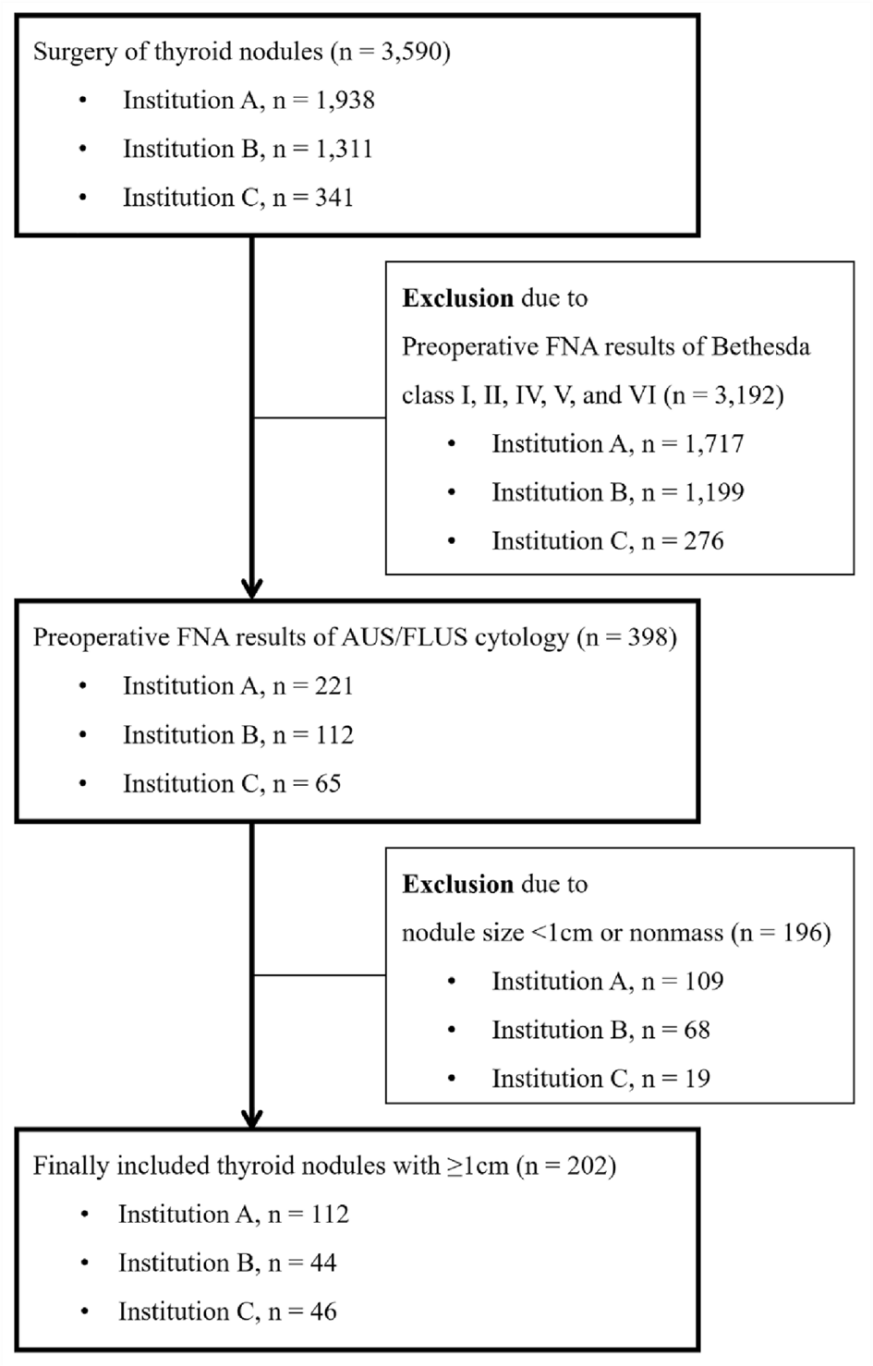
Diagram of the study group which included patients from 3 different hospitals. *FNA* fine-needle aspiration, *AUS* atypia of undetermined significance, *FLUS* follicular lesion of undetermined significance.

We collected 3,590 consecutive patients who underwent thyroid surgery at each hospital (Institution A, Jan 2014 to Jun 2019, n = 1938; Institution B, Jan 2019 to Sep 2019, n = 1311; and Institution C, Jan 2017 to Jun 2019, n = 341; Fig. 2). In these patients, we searched for nodules ≥ 1 cm that were confirmed as Bethesda category III on FNA and surgically excised. Finally, 202 nodules in 202 patients were included in this study (A, n = 112; B, n = 44; and C, n = 46; Fig. 2).

### US examinations and imaging interpretation

**Figure 3:**
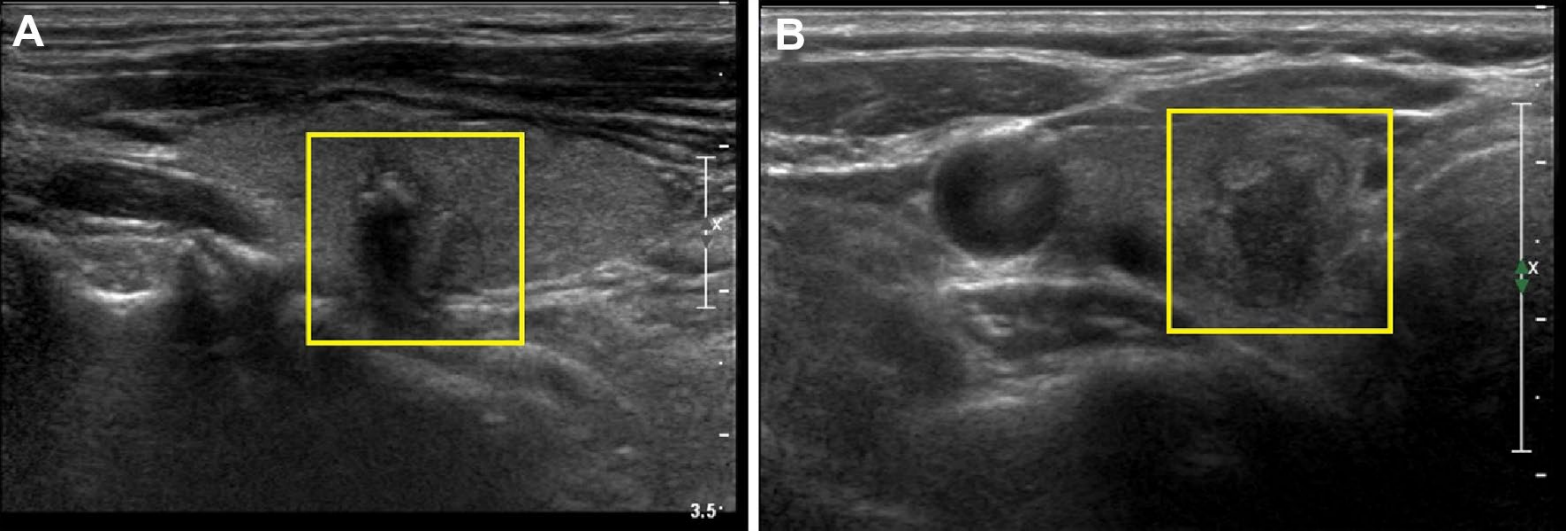
Deep convolutional neural network (CNN) processing using ultrasonography (US) images of malignant thyroid nodules with atypia of undetermined significance (AUS, **A**) or follicular lesion of undetermined significance (FLUS, **B**) results on fine-needle aspiration (FNA). (**A**) A captured thyroid US image of a yellow square region-of-interest covering the whole thyroid nodule in a 71-year-old man. There was a 10 mm-sized thyroid nodule diagnosed as AUS on US-guided FNA. The cancer probability calculated by CNN was 90.9%. The patient underwent surgery, and pathology confirmed papillary carcinoma. (**B**) A captured thyroid US image of a yellow square region-of-interest covering the whole nodule in a 57-year-old woman. There was a 12 mm-sized thyroid nodule diagnosed as FLUS on US-guided FNA. The cancer probability calculated by CNN was 88.1%. The patient underwent surgery, and pathology confirmed encapsulated angioinvasive follicular carcinoma.

US examinations were performed using several types of US machines (Supplementary Information 1). One clinician at each hospital reviewed the preoperative thyroid US images, selected the most representative image of each thyroid nodule, and saved them as JPEG files (Fig. 3). A square region-of-interest (ROI) was then drawn to cover each whole nodule using the Microsoft Paint program (version 6.1; Microsoft Corporation, Redmond, WA, USA). The saved images from the 3 hospitals were randomly mixed and numbered by an experienced radiologist (Fig. 3). They were independently reviewed by the following 8 physicians, none who had information on the cytopathologic results of each thyroid nodule: 2 faculty radiologists (7 and 10 years of experience in thyroid imaging), 2 less experienced radiologists (2 and 4 years of experience), 2 faculty endocrinologists (more than 5 years of experience), and 2 less experienced endocrinologists (1 year of experience). Before reviewing the captured images, all of 8 physicians were trained using the user’s guide by ACR TI-RADS^28^.

The 8 physicians evaluated the following US features using the TI-RADS system proposed by the ACR^28^: composition (cystic or almost completely cystic, spongiform, mixed cystic and solid, solid or almost completely solid), echogenicity (anechoic, hyperechoic or isoechoic, hypoechoic, very hypoechoic), shape (wider-than-taller, taller-than-wide), margin (smooth, ill-defined, lobulated or irregular, extrathyroidal extension), and echogenic foci (none or large comet-tail artifacts, macrocalcifications, peripheral calcifications, punctate echogenic foci). Eight physicians determined malignancy risk using the ACR TI-RADS system and the assigned risk levels ranged from TI-RADS (TR) 1 (benign, 0 points), TR2 (not suspicious, 2 points), TR3 (mildly suspicious, 3 points), TR4 (moderately suspicious, 4–6 points), to TR5 (highly suspicious, 7 or more points) (Supplementary Table 2)^28^.

### Deep convolutional neural network

In this study, we used a computer-aided diagnosis (CAD) program to differentiate malignancy from benign lesions, which was recently developed with 13,560 US images of thyroid nodules using a deep convolutional neural network^14^. The CAD program was based on a transfer learning technique equipped with fine-tuning in order to overcome the limited amount of data and maximize accuracy through a combination of big data and deep learning. Four sophisticated pre-trained nets (AlexNet, SqueezeNet, GoogLeNet, and Inception-ResNet-v2) were used and a weighted average process was performed (see Supplementary Information 2 and Supplementary Fig. 1 for details on the averaging process). To train the networks with the fine-tuning process, the stochastic gradient descent method with momentum was used as a solver, and various parameter values (initial learning rate, learning rate dropping periods, max epochs, mini-batch sizes, etc.) were chosen through a selection process including Bayesian optimization.

### Statistical analysis

We collected data on the final diagnosis of each thyroid nodule after surgery that had been recorded in the electronic medical records of each hospital. Cancer probabilities were calculated using CNN, and were presented as percentages (0–100%). Categorical data were summarized as frequencies and percentages, and continuous variables were presented as means ± standard deviations or median (interquartile range). The Shapiro–Wilk test was performed to assess the normality of continuous variables. We evaluated differences in variables using the independent two-sample t-test, Mann–Whitney U test, Chi-square test, or Fisher’s exact test.

Sensitivities and specificities of the 8 physicians and CNN for predicting malignancy were evaluated and compared by generalized estimating equation (GEE). Of the risk levels of the ACR TI-RADS system, we used a cut-off point of TR 5 for the 8 physicians. The cut-off values of CNN were determined with Youden’s index. A receiver operating characteristic (ROC) curve analysis and areas under the curve (AUCs) were compared by DeLong’s test. The diagnostic performances of the 8 physicians and CNN were evaluated in each AUS and FLUS group, and also compared using the ROC curve analysis.

We evaluated interobserver variability among all 8 physicians using Fleiss’ Kappa, and then divided the physicians into 2 groups to also compare interobserver variability among the 4 radiologists and among the 4 endocrinologists separately with Fleiss’ Kappa. A kappa value (k) of less than 0 indicated no agreement; 0–0.20, slight agreement; 0.21–0.40, fair agreement; 0.41–0.60, moderate agreement; 0.61–0.80, substantial agreement; and 0.81–1.00, almost perfect agreement^29^.

All *P* values were calculated using the two-tailed t-test and a *P* < 0.05 was considered to indicate statistical significance. All statistical analyses were performed using SAS software, version 9.4 (SAS Institute, Inc., Cary, NC, USA) and R Core Team (2020) (R: A language and environment for statistical computing. R Foundation for Statistical Computing, Vienna, Austria. URL https://www.R-project.org/)**.**

## Supplementary Information

Supplementary Information 1.
