# Supplementary material for: Diagnosing thyroid nodules with atypia of undetermined significance/follicular lesion of undetermined significance cytology with the deep convolutional neural network
Source: Sci Rep. 2021 Oct 8;11:20048. doi: 10.1038/s41598-021-99622-0 (PMC8501016; doi:10.1038/s41598-021-99622-0)
Supplement: Supplementary file 1 — Supplementary Information 1. [file 41598_2021_99622_MOESM1_ESM.docx]

**Supplementary Material**

**Using the deep convolutional neural network to evaluate thyroid nodules with atypia of undetermined significance/follicular lesion of undetermined significance cytology: Multicenter study**

Inyoung Youn, Eunjung Lee, Jung Hyun Yoon, Hye Sun Lee, Mi-Ri Kwon, Juhee Moon, Sunyoung Kang, Seul Ki Kwon, Kyong Yeun Jung, Young Joo Park, Do Joon Park, Sun Wook Cho, Jin Young Kwak

**Correspondence:** docjin@yuhs.ac (J.Y.K.), swchomd@snu.ac.kr (S.W.C.)

**Table of contents**

Supplementary Information 1 2

Supplementary Information 2 3

Supplementary Table 1 4

Supplementary Table 2 6

Supplementary Figure 1 7

**Supplementary Information 1.** Information about the ultrasonography machines in each hospital.

Institution A used one of the following 4 machines: Aixplorer (SuperSonic Imagine, Aix-en-Provence, France), iU22 (Philips Healthcare, Bothell, WA), EPIQ 7G (Philips Healthcare, Bothell, WA), and LOGIQ E9 (GE Healthcare, Milwaukee, WI, USA). Institution B used the iU22 (Philips Healthcare, Bothell, WA) or EPIQ 7G (Philips Healthcare, Bothell, WA). Institution C used the LOGIQ7 (GE Healthcare, Milwaukee, WI, USA) or Affinity 50G (Philips Healthcare, Bothell, WA). Each system was equipped with a linear, high-frequency transducer (5-14MHz).

**Supplementary Information 2.** Deep CNN protocol.

The convolutional neural network (CNN), one of the popular deep learning structures, is now widely used in medical imaging. In general, a good learning process requires big data that is often not available in the medical imaging field. For this reason, we use CNN models trained with huge amounts of common data with various classes and modify these sophisticated nets to fit the given medical data. This process is called transfer learning (reusing the pre-trained network) and fine-tuning (taking weights of a pre-trained network and using them for the initialization of the model being trained with new data). Each net has a distinct structure that extracts features from data and it uses these features to determine the final recommendation. Different structures can see different aspects in data that can lead to various conclusions. To increase the reliability of the results acquired with CNNs, we selected four pre-trained nets (AlexNet, SqueezeNet, GoogLeNet, Inception-ResNet-v2) and use the AUCs as weights during averaging to combine the results from each net (see Supplementary Figure 1 for better understanding). In the training process, 10% of the training data are randomly chosen as the validation set and AUCs are obtained from this validation process. The deployed results from each net are in the form of probability values. These probability values corresponding to each category are gathered and used to make the final decision.

**Supplementary Table 1.** Summary of US points for the ACR TI-RADS scores assigned by each of the 8 physicians.

|  | **Benign** | | | | | | | | | **Malignant** | | | | | | | |
| --- | --- | --- | --- | --- | --- | --- | --- | --- | --- | --- | --- | --- | --- | --- | --- | --- | --- |
|  | **R1** | **R2** | **R3** | **R4** | | **E1** | **E2** | **E3** | **E4** | **R1** | **R2** | **R3** | **R4** | **E1** | **E2** | **E3** | **E4** |
| Composition | | | | | | | | | |  | | | | | | | |
| 0 point^a^ | 2 | 1 | 1 | | 0 | 0 | 0 | 1 | 0 | 4 | 5 | 1 | 2 | 1 | 0 | 1 | 0 |
| 1 point^b^ | 17 | 42 | 13 | | 12 | 5 | 7 | 35 | 7 | 19 | 28 | 15 | 14 | 6 | 4 | 37 | 10 |
| 2 points^c^ | 67 | 43 | 72 | | 74 | 81 | 79 | 50 | 79 | 93 | 83 | 100 | 100 | 109 | 112 | 78 | 106 |
| Echogenicity | | | | | | | | | |  | | | | | | | |
| 0 point^d^ | 2 | 1 | 1 | | 0 | 0 | 0 | 0 | 0 | 4 | 2 | 0 | 1 | 1 | 0 | 1 | 0 |
| 1 point^e^ | 56 | 34 | 42 | | 54 | 47 | 49 | 48 | 45 | 59 | 23 | 46 | 59 | 42 | 49 | 52 | 41 |
| 2 points^f^ | 26 | 43 | 42 | | 24 | 35 | 32 | 37 | 36 | 41 | 76 | 65 | 33 | 68 | 62 | 62 | 65 |
| 3 points^g^ | 2 | 8 | 1 | | 8 | 4 | 5 | 1 | 5 | 12 | 15 | 5 | 23 | 5 | 5 | 1 | 10 |
| Shape | | | | | | | | | |  | | | | | | | |
| 0 point^h^ | 84 | 77 | 82 | | 85 | 79 | 85 | 86 | 85 | 102 | 85 | 99 | 103 | 96 | 108 | 110 | 106 |
| 3 points^i^ | 2 | 9 | 4 | | 1 | 7 | 1 | 0 | 1 | 14 | 31 | 17 | 13 | 20 | 8 | 6 | 10 |
| Margin | | | | | | | | | |  | | | | | | | |
| 0 point^j^ | 82 | 79 | 69 | | 81 | 73 | 83 | 79 | 65 | 75 | 61 | 52 | 75 | 68 | 74 | 70 | 54 |
| 2 points^k^ | 4 | 7 | 16 | | 5 | 6 | 3 | 7 | 17 | 34 | 51 | 53 | 39 | 30 | 37 | 43 | 55 |
| 3 points^l^ | 0 | 0 | 1 | | 0 | 7 | 0 | 0 | 4 | 7 | 4 | 11 | 2 | 18 | 5 | 3 | 7 |
| Echogenic foci (Choose all that apply) | | | | | | | | | |  | | | | | | | |
| 0 point^m^ | 72 | 80 | 71 | | 65 | 58 | 58 | 79 | 74 | 52 | 66 | 53 | 50 | 45 | 46 | 61 | 61 |
| 1 point^n^ | 3 | 4 | 5 | | 3 | 4 | 4 | 5 | 4 | 9 | 18 | 16 | 3 | 4 | 4 | 22 | 12 |
| 2 points^o^ | 1 | 2 | 2 | | 3 | 8 | 4 | 1 | 1 | 10 | 6 | 8 | 9 | 16 | 14 | 10 | 5 |
| 3 points^p^ | 9 | 0 | 7 | | 15 | 16 | 20 | 1 | 7 | 36 | 26 | 34 | 38 | 51 | 52 | 23 | 38 |
| 4 points^q^ | 1 | 0 | 0 | | 0 | 0 | 0 | 0 | 0 | 6 | 0 | 4 | 9 | 0 | 0 | 0 | 0 |
| 5 points^r^ | 0 | 0 | 1 | | 0 | 0 | 0 | 0 | 0 | 2 | 0 | 1 | 5 | 0 | 0 | 0 | 0 |
| 6 points^s^ | 0 | 0 | 0 | | 0 | 0 | 0 | 0 | 0 | 1 | 0 | 0 | 2 | 0 | 0 | 0 | 0 |

^a^Cystic, almost completely cystic, spongiform. ^b^Mixed cystic and solid. ^c^Solid, almost completely solid. ^d^Anechoic. ^e^Hyperechoic, Isoechoic. ^f^Hypoechoic. ^g^Very hypoechoic. ^h^Wider-than-tall. ^i^Taller-than-wide. ^j^Smooth, ill-defined. ^k^Lobulated, irregular. ^l^Extra-thyroidal extension. ^m^No, Comet-tail artifact. ^n^Macrocalcification. ^o^Rim calcification. ^p^Microcalcification, Macrocalcification with Rim calcification. ^q^Macrocalcification with Microcalcification. ^r^Rim calcification with Microcalcification. ^s^Macrocalcification with Rim calcification and Microcalcification.

Abbreviations: US = ultrasonography; ACR = The American Society of Radiology; TI-RADS = Thyroid Imaging Reporting and Data System; R = radiologist; E = endocrinologist.

**Supplementary Table 2.** Comparison of the number of TR scores assigned by the 8 physicians.

|  | **Benign** | | | | | | | | **Malignant** | | | | | | | |
| --- | --- | --- | --- | --- | --- | --- | --- | --- | --- | --- | --- | --- | --- | --- | --- | --- |
|  | **R1** | **R2** | **R3** | **R4** | **E1** | **E2** | **E3** | **E4** | **R1** | **R2** | **R3** | **R4** | **E1** | **E2** | **E3** | **E4** |
| TR1 | 2 | 1 | 1 | 0 | 0 | 0 | 0 | 0 | 3 | 4 | 0 | 2 | 1 | 0 | 1 | 0 |
| TR2 | 10 | 17 | 6 | 5 | 1 | 4 | 23 | 3 | 6 | 13 | 6 | 6 | 0 | 1 | 12 | 4 |
| TR3 | 35 | 28 | 26 | 39 | 23 | 27 | 32 | 33 | 22 | 11 | 19 | 17 | 13 | 15 | 23 | 15 |
| TR4 | 36 | 36 | 44 | 34 | 46 | 46 | 30 | 39 | 41 | 36 | 36 | 33 | 43 | 54 | 52 | 48 |
| TR5 | 3 | 4 | 9 | 8 | 16 | 9 | 1 | 11 | 44 | 52 | 55 | 58 | 59 | 46 | 28 | 49 |
| AUS (n=158) | | | | | | | | |  | | | | | | | |
| TR1 | 2 | 1 | 1 | 0 | 0 | 0 | 0 | 0 | 3 | 3 | 0 | 2 | 1 | 0 | 1 | 0 |
| TR2 | 7 | 15 | 3 | 3 | 0 | 3 | 15 | 2 | 5 | 11 | 6 | 5 | 0 | 1 | 11 | 4 |
| TR3 | 19 | 13 | 17 | 21 | 12 | 13 | 20 | 18 | 18 | 9 | 15 | 14 | 11 | 12 | 22 | 13 |
| TR4 | 20 | 20 | 22 | 18 | 26 | 27 | 14 | 21 | 39 | 34 | 33 | 30 | 39 | 49 | 47 | 43 |
| TR5 | 2 | 1 | 7 | 8 | 12 | 7 | 1 | 9 | 43 | 51 | 54 | 57 | 57 | 46 | 27 | 48 |
| FLUS (n=44) | | | | | | | | |  | | | | | | | |
| TR1 | 0 | 0 | 0 | 0 | 0 | 0 | 0 | 0 | 0 | 1 | 0 | 0 | 0 | 0 | 0 | 0 |
| TR2 | 3 | 2 | 3 | 2 | 1 | 1 | 8 | 1 | 1 | 2 | 0 | 1 | 0 | 0 | 1 | 0 |
| TR3 | 16 | 15 | 9 | 18 | 11 | 14 | 12 | 15 | 4 | 2 | 4 | 3 | 2 | 3 | 1 | 2 |
| TR4 | 16 | 16 | 22 | 16 | 20 | 19 | 16 | 18 | 2 | 2 | 3 | 3 | 4 | 5 | 5 | 5 |
| TR5 | 1 | 3 | 2 | 0 | 4 | 2 | 0 | 2 | 1 | 1 | 1 | 1 | 2 | 0 | 1 | 1 |

TR = risk level of the Thyroid Imaging Reporting and Data System; R = radiologist; E = endocrinologist; AUS = atypia of undetermined significance; FLUS = follicular lesion of undetermined significance.

| Deep CNNs | Probability values | | AUC |
| --- | --- | --- | --- |
|  | ( $p_{j1}+p_{j2}=1 )$ | | $( 0\leq A_{j}\leq1 )$ |
| **AlexNet** | $p_{11}$ | $p_{12}$ | $A_{1}$ |
| **SqueezeNet** | $p_{21}$ | $p_{22}$ | $A_{2}$ |
| **GoogLeNet** | $p_{31}$ | $p_{32}$ | $A_{3}$ |
| **Inception-ResNet-v2** | $p_{41}$ | $p_{42}$ | $A_{4}$ |
| Final decision is made based on the values $P_{1}$ and $P_{2}$ such that  $P_{1}=\sum_{j=1}^{4} p_{j1}\cdot w_{j} \mathrm{and} P_{2}=\sum_{j=1}^{4} p_{j2}\cdot w_{j},$  where $w_{j}=A_{j}/(A_{1}+A_{2}+A_{3}+A_{4})$ for $j=1,\cdots, 4.$ | | | |

**Supplementary Figure 1.** Structure of deep CNN used in this study.
